# Editorial: Global excellence in plant science: Southeast Asia

**DOI:** 10.3389/fpls.2023.1285250

**Published:** 2023-09-08

**Authors:** Febri Doni, Muhamad Shakirin Mispan

**Affiliations:** ^1^ Department of Biology, Faculty of Mathematics and Natural Sciences, Universitas Padjadjaran, West Java, Indonesia; ^2^ Institute of Biological Sciences, Faculty of Science, Universiti Malaya, Kuala Lumpur, Malaysia

**Keywords:** sustainable crop production, system of rice intensification, plant-microbe interactions, plant genomics, disease resistance, abiotic stress resistance, photosynthesis, beneficial microbes

## Introduction

The pivotal role of plant science research lies in elevating food and nutrition security, fostering ecosystem sustainability, driving economic growth, and promoting social equity for the future (Dhankher and Foyer, 2018). Plant scientists employ innovative techniques to unravel plant biology’s complexities, genetics, evolution, and physiology, leading to the development of robust, high-yield crop varieties suited to regional challenges (Cheng et al., 2023). This Research Topic aims to highlight recent plant science advancements and anticipate upcoming challenges, with a focus on Southeast Asia’s scientific progress in this field.

## Enhancing production and biotic and abiotic stresses resistance of cultivated rice

Rice stands as a vital global crop, catering to over half of the world’s population and particularly prominent in Asia, where it is both the largest producer and consumer (Akbari et al., 2023). However, the projected 45% increase in global rice consumption by 2050 due to population growth raises concerns about food scarcity and security (FAO, 2020). The challenge lies in boosting rice yield sustainably, as increasing cultivation area is not feasible (Thakur et al., 2023).

Doni et al. highlighted the positive performance of the system of rice intensification (SRI) methods incorporated with *Trichoderma*-rice plant symbiosis in increasing rice growth and yield. The results indicated that certain effects of SRI management elicit beneficial changes in rice cells’ gene expression. This method can serve as a potential solution, altering rice plants’ productivity by enhancing soil conditions, organic matter, and reducing agrochemicals. However, more systematic studies combining the effects of all the components together comparing with the standard rice production system are essential.

Furthermore, salinity stress further threatens rice yield and quality (Liu et al., 2022). Open field experiments by Li et al. investigated the effects of salinity across rice’s growth stages, revealing that elevated sodium (Na^+^) levels impede growth and hinder nitrogen assimilation. Emphasis on field management during the vulnerable panicle initiation stage is crucial in saline-prone regions to mitigate salt-induced stress, safeguarding rice plants and ensuring proper freshwater irrigation for subsequent growth cycles.

On another note, sheath blight disease caused by the soil-borne pathogenic fungus *Rhizoctonia solani* Kuhn, constitutes the most detrimental fungal infection affecting rice (Senapati et al., 2022). Hossain et al. focused on enhancing resistance in *Oryza sativa* pv. UKMRC2 by developing varietal resistance against *R. solani*. The study revealed that the combined presence of certain alleles, RM224, RM27360, and K39512, exhibited heightened disease resistance compared to individual alleles. This established validation of the qSBR11-1TT QTL within the UKMRC2 genetic context, demonstrating potential for future sheath blight resistance breeding. The integration of marker-assisted selection programs emerges as a promising strategy to enhance resistance in rice varieties.

## Physiological traits and yield of maize are increased by intercropping with blackgram

Photosynthesis, crop vitality, and the allocation of dry matter are pivotal factors that significantly impact both crop productivity and quality (Sherin et al., 2022). Varatharajan et al. investigated the impact of integrated crop management (ICM) modules and cropping systems on maize, revealing that the conservation agriculture-based ICM module with blackgram intercropping notably enhanced physiological traits, photosynthetic rate, and productivity. This technology package holds promise for boosting maize production in Asia and presents a broader avenue for ICM implementation in various crops, contributing to enhanced productivity and sustainability. The study’s insights emphasize the positive effects of ICM modules and legume interventions on both sole and intercropped maize, further supporting their adoption for increased agricultural productivity.

## Improving banana resistance towards *Fusarium oxysporum* f. sp. *cubense*

Bananas hold immense importance for developing nations, particularly in Southeast Asia (Permadi et al., 2023) and Fusarium wilt infestation is significantly impacting banana production (Pegg et al., 2019). Abdullah et al. elucidated the critical role of vacuolar processing enzyme (VPE)-mediated programmed cell death (PCD) in modulating susceptibility responses during compatible interactions, facilitating colonization of the banana host plant by *Fusarium oxysporum* f. sp. *cubense* tropical race 4 (*FocTR4*). A total of seven *MaVPEs* genes were identified, revealing a sequence of cellular events intricately linked to *MaVPEs* and their signalling counterparts. *FocTR4* infection increased reactive oxygen species (ROS) activity, correlating with elevated *MaVPE* activity. These insights shed light on banana plants’ mechanism during *FocTR4* infection, particularly VPE-mediated PCD. Future prospects include using gene editing tools like CRISPR to regulate *MaVPE* activities, potentially enhancing strategies to protect bananas against *FocTR4* infection.

## Promoting *Elaeis guineensis* Jacq. growth through *in vitro* biotization with beneficial microbes

Oil palm (*Elaeis guineensis* Jacq.) is a primary global oil-producing crop, supplying around 40% of traded vegetable oil (Murphy et al., 2021). Tissue culture techniques are commonly employed for oil palm propagation, yet progress has been impeded by prolonged culture duration and limited yield of callus and embryogenic callus, hindering high-quality plantlet production (Weckx et al., 2019). To address this, Lim et al. in their mini-review proposed biotization using plant growth promoting rhizobacteria (PGPRs) to enhance oil palm growth during tissue culture micropropagation. PGPRs can stimulate quorum-sensing signals, promoting plant cell development, and induce biochemical and molecular changes. Biotization with beneficial microbes during the embryogenic stage holds promise for improving subsequent plant growth cycles.

## Biodiversity, origin, and evolution of plants in Asia

Asia, renowned for its vast size and ecological diversity, hosts one of the world’s highest plant diversity levels, establishing it as a crucial biodiversity hub (Tan et al., 2022). Le et al. conducted a study in Vietnam to explore the genetic diversity of *Panax vietnamensis* Ha & Grushv., an endemic and endangered ginseng species. Using the Angiosperm-353 probe set, they examined 353 low-copy nuclear genes in *P. vietnamensis*. The findings revealed a single panmictic population across recognized wild and cultivated *P. vietnamensis* groups, with origins in two ancestral groups and varying mixture levels. Notably, the observed genetic diversity and gene flow were attributed to human activities, including diverse cultivation practices and regional trade.

Another interesting study was conducted by Ye et al. to analyse floral evolution in East Asia through meta-analyses of radiation dates (crown ages) of endemic seed plant genera. The study particularly emphasized the potential influence of monsoon intensification on the evolution of East Asian flora. The findings indicated that the enhancement of the monsoon in East Asia plays a role in driving the evolution of its flora. Nevertheless, there is still ample opportunity for further studies that incorporate genomic data, extensive geographic sampling, and an exploration of the tempo-spatial evolution of endemic genera.

## Conclusion

This Research Topic showcases an array of scientific efforts focusing on current important aspects of plant science, resonating not just within Southeast Asia but on a worldwide canvas as well. These findings introduce innovative methods and ideas that could have positive effects beyond certain crops and regions, kindling prospects to improve global food security. However, it is important to acknowledge the need for more research efforts and initiatives to further advance plant science in Southeast Asia.

## Author contributions

FD: Writing – original draft, Writing – review & editing. MM: Writing – original draft, Writing – review & editing.
